# Integrated silicon photonic MEMS

**DOI:** 10.1038/s41378-023-00498-z

**Published:** 2023-03-20

**Authors:** Niels Quack, Alain Yuji Takabayashi, Hamed Sattari, Pierre Edinger, Gaehun Jo, Simon J. Bleiker, Carlos Errando-Herranz, Kristinn B. Gylfason, Frank Niklaus, Umar Khan, Peter Verheyen, Arun Kumar Mallik, Jun Su Lee, Moises Jezzini, Iman Zand, Padraic Morrissey, Cleitus Antony, Peter O’Brien, Wim Bogaerts

**Affiliations:** 1 École Polytechnique Fédérale de Lausanne (EPFL), 1015 Lausanne, Switzerland; 2 School of Aerospace, Mechanical and Mechatronic Engineering, The University of Sydney, Camperdown, NSW 2006 Australia; 3 Swiss Center for Electronics and Microtechnology (CSEM), 2002 Neuchâtel, Switzerland; 4 Division of Micro and Nanosystems, KTH Royal Institute of Technology, 114 28 Stockholm, Sweden; 5 Department of Information Technology, Photonics Research Group, Ghent University - IMEC, Technologiepark-Zwijnaarde 126, 9052 Gent, Belgium; 6 imec vzw. 3DSIP Department, Si Photonics Group, Kapeldreef 75, 3001 Leuven, Belgium; 7 Tyndall National Institute, Lee Maltings Complex Dyke Parade, Cork, T12 R5CP Ireland

**Keywords:** Other photonics, Nanoscale devices, NEMS

## Abstract

Silicon photonics has emerged as a mature technology that is expected to play a key role in critical emerging applications, including very high data rate optical communications, distance sensing for autonomous vehicles, photonic-accelerated computing, and quantum information processing. The success of silicon photonics has been enabled by the unique combination of performance, high yield, and high-volume capacity that can only be achieved by standardizing manufacturing technology. Today, standardized silicon photonics technology platforms implemented by foundries provide access to optimized library components, including low-loss optical routing, fast modulation, continuous tuning, high-speed germanium photodiodes, and high-efficiency optical and electrical interfaces. However, silicon’s relatively weak electro-optic effects result in modulators with a significant footprint and thermo-optic tuning devices that require high power consumption, which are substantial impediments for very large-scale integration in silicon photonics. Microelectromechanical systems (MEMS) technology can enhance silicon photonics with building blocks that are compact, low-loss, broadband, fast and require very low power consumption. Here, we introduce a silicon photonic MEMS platform consisting of high-performance nano-opto-electromechanical devices fully integrated alongside standard silicon photonics foundry components, with wafer-level sealing for long-term reliability, flip-chip bonding to redistribution interposers, and fibre-array attachment for high port count optical and electrical interfacing. Our experimental demonstration of fundamental silicon photonic MEMS circuit elements, including power couplers, phase shifters and wavelength-division multiplexing devices using standardized technology lifts previous impediments to enable scaling to very large photonic integrated circuits for applications in telecommunications, neuromorphic computing, sensing, programmable photonics, and quantum computing.

## Introduction

Photonic integration holds promise for combining complex optical systems onto a single chip, and over the past decades, significant progress towards this goal has been achieved^1–3^. Several material and technology platforms have reached industrial maturity. Among the most widely adopted technology platforms are planar lightwave circuits (PLCs)^4,5^, indium phosphide (InP)^6^, silicon nitride (SiN)^7,8^, and silicon photonics^9^. Research further explores additional material platforms, such as lithium niobate^10,11^, aluminium nitride^12,13^ and single crystal diamond^14^, by virtue of the material’s high electro-optic coefficient, strong piezo-electric effect, or potential to host colour centres as single photon sources, respectively. Although these efforts are of high potential and interest, particularly for quantum photonics^15^, significant progress is still required to achieve industrial maturity^16^.

The choice of a material and technology platform is driven by specific application requirements^15^. For example, fibre-optical telecommunications has been a driver of near-infrared photonic integrated circuits, such as integrated arrayed waveguide gratings that are primarily built in planar lightwave circuits^17,18^. These low absorption and high-quality waveguides enable very low loss, yet the low-index difference of the silica waveguides leads to relatively large bend radii and thus limits further miniaturization. In addition, PLC technology provides only passive photonic components. Silicon nitride waveguides have recently gained interest due to their significant advancement in reducing on-chip propagation losses and ability to support wavelengths in the visible^8^ and near-infrared range for imaging applications; however, current silicon nitride technology platforms only support passive elements. Conversely, indium phosphide-based integrated photonics allow for the integration of active components, including laser sources and photodetectors alongside waveguides and passive components^19^. However, III–V semiconductor wafers remain expensive and limited in size, hitherto prohibiting cost-efficient very large-scale integration.

In comparison, silicon photonics are distinct due to their ability to leverage mature high-volume manufacturing processes and build on the immense knowledge base of the microelectronics semiconductor industry^20,21^. Consequently, research and development in silicon photonics has accelerated over the past decade: the propagation loss of passive silicon waveguides has been drastically reduced to reach 0.04 dB/cm^22^, electro-optic modulators based on the plasma-dispersion effect exceed a 50 GHz bandwidth^23^, thermo-optic phase shifters have been integrated for tuning^24^ and ultrafast epitaxially grown germanium photodetectors^25^ have been included on a single substrate and all are available in 200 or 300 mm wafer processes offered by foundries today^26,27^. In addition, advanced process design kits, equivalent to their well-known counterparts offered by foundries for microelectronic integrated circuits, are emerging with numerous optimizations specifically for photonics^28^. The availability of dedicated design kits allows circuit designers to focus on increasingly complex circuit-level design to achieve higher levels of functionality.

The main drivers for silicon photonics today are high-speed transceivers exceeding 400 Gb/s for datacentres^29^. While fabricating such transceivers on a few square millimetres represents a technological milestone, the capability of silicon photonics goes far beyond transceivers; indeed, several far-reaching novel photonic concepts have recently been discussed. A first example is programmable photonic integrated circuits (PICs)^30^, also referred to as field-programmable photonic integrated circuits (FPPICs) or reconfigurable photonics. Such programmable PICs are composed of a network of fundamental linear photonic circuit elements, i.e., phase shifters and power couplers, that can be reconfigured or programmed to fulfil a variety of functions. Conceptually, this can be compared to field-programmable gate arrays (FPGA) in microelectronics, where a single physical circuit can be reprogrammed many times to perform different functions. A second example consists of photonic accelerators or photonic tensor cores^31^, where a network of phase shifters and power couplers performs vector-matrix multiplication in the optical domain^32^. Third, neuromorphic computing units can be implemented in photonics and provide an avenue to efficiently solve complex combinatorial problems^33^. Finally, quantum photonic integrated circuits are predicted to be an enabling technology system for quantum computing that benefit exponentially from scaling^34^. Today, programmable photonics, photonic accelerators, photonic neural networks and quantum photonics are commercially investigated at small to medium scales^35^, driving the need for enhancement, scaling and maturing of PIC technology.

To realize their full potential, these emerging concepts require advanced very large-scale PICs, with tens or potentially hundreds of thousands of individual phase shifters and power couplers combined in a single photonic integrated circuit. In today’s standard technologies in state-of-the-art silicon photonics foundries, phase shifters can be implemented based on thermo-optic tuning with low-loss and compact footprint, and power couplers can be implemented using plasma dispersion effect-based fast Mach‒Zehnder interferometer (MZI) modulators. However, thermo-optic phase shifters consume several mW per device, with limited integration density due to thermal cross-talk^24,36^, and due to the weak electro-optic effect, the length of electro-optic MZI modulators is typically hundreds of µm^37^. Alternative approaches explore ring-resonator-based modulators, which provide smaller footprints but have a narrow spectral response and are prone to manufacturing variations, thus requiring additional phase shifters for wavelength tuning^38^. Plasmonic–organic hybrid devices have also recently shown high potential^39^, yet losses in these devices remain significant, and demonstration of very large-scale integration currently remains elusive.

Evidently, these technology implementations do not fulfil the requirements for very large-scale PICs, which impose extremely stringent requirements on the performance of individual components. Namely, phase shifters and power couplers are required to be broadband, exhibit low optical loss, have low power consumption, have a short optical path length and require sufficiently fast response time for efficient reconfiguration. Moreover, the actuation mechanisms need to be compatible with large-scale fabrication and dense integration.

**Fig. 1:**
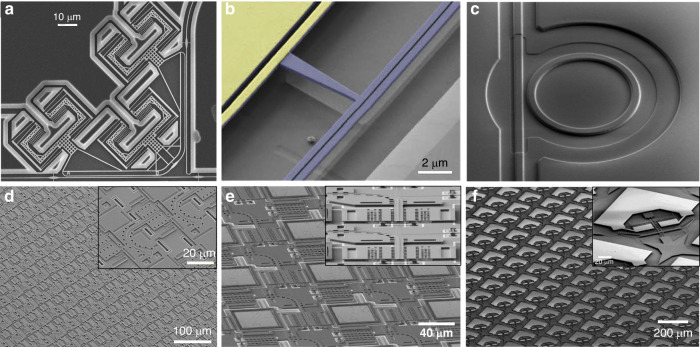
Representative examples of recently demonstrated silicon photonic MEMS. Individual components based on dedicated processes on silicon-on-insulator wafers include **a** tunable couplers^44^, **b** phase shifters^42^, and **c** ultrasound sensors^50^, and silicon photonic MEMS based on specifically developed microfabrication processes using multiple layers have shown **d** beam steering for 3D imaging^41^ and **e**, **f** large-scale photonic switch matrices for optical circuit switching in datacentres^40,46^. (Permissions: **a** Adapted with permission from ref. ^44^ © The Optical Society. **b** Adapted with permission from ref. ^42^ © The Optical Society. **c** Adapted with permission from ref. ^50^ © Springer Nature. **d** Adapted from ref. ^41^ under creative commons license CC BY 4.0. **e** Adapted with permission from ref. ^40^ © The Optical Society. **f** Adapted from ref. ^46^ under creative commons license CC BY 4.0)

Silicon photonic microelectromechanical systems (MEMS) technology can simultaneously overcome all these limitations. For example, one of the largest-scale PICs today, a 240 × 240 silicon photonic switch matrix with 57,600 individual switching elements, has been demonstrated experimentally by combining MEMS with silicon photonic waveguides^40^. MEMS itself is a mature technology, and billions of MEMS micromirrors, microphones, accelerometers, gyroscopes, etc., reliably power countless consumer electronic devices. The combination of MEMS with integrated photonics, however, requires specific engineering and process integration. The recently demonstrated large-scale silicon photonic MEMS switch matrix^40^ and large-scale silicon photonic MEMS LiDAR^41^ clearly show the potential of the combination of silicon photonics and MEMS. Yet this specific technology requires multilayer stacking of waveguides, and the involved high-temperature deposition processes make it challenging to be compatible with standard silicon photonic components such as high-speed modulators and photodiodes. The potential of silicon photonic MEMS has also been recognized in early work that focuses on demonstrations of individual devices. For example, phase shifters^42,43^, switches^44–46^, tunable ring resonators^47,48^, optomechanical photonic crystal cavities^49^ and compact optomechanical ultrasound sensors^50^ have been demonstrated as individual devices or small-scale circuits in single-layer silicon photonics. Figure 1 summarizes a selection of hitherto experimentally demonstrated individual silicon photonic MEMS devices (Fig. 1a–c) and a selection of recently demonstrated scaled silicon photonic MEMS for beam steering (Fig. 1d) and switching in datacentres (Fig. 1e, f). A more complete account of previously demonstrated silicon photonic MEMS can be found in a recent review^51^.

To leverage the full potential of MEMS in integrated photonics, it is essential to integrate MEMS with standardized silicon photonics technology. A typical standardized silicon photonics platform consists of a thin device layer (150–400 nm, with 220 nm as a commonly used thickness) in a silicon-on-insulator (SOI) wafer that is separated from the handle layer by a buried oxide layer (2–3 μm). The device layer is structured by a full layer thickness etch (and potentially one or several partial layer thickness etches) to form waveguiding structures, and the buried oxide prevents optical power leakage to the substrate. Several optimized doping levels in the device layer allow for modulator integration, germanium epitaxial growth and associated doping for photodiode integration. A back-end-of-line (BEOL) stack can include several metallization routing layers and intermetal dielectrics that serve as both electrical isolation and optical cladding. The electrical interfaces are accessible by a final bond pad metallization layer and the optical interfaces are exposed by a partial removal of the BEOL stack for optimum optical coupling. Access to the waveguide layer can be provided by a dedicated etch through the BEOL material stack. Technical details of several open access standard platforms following the above technical concepts have been published over the past decade^26^. Our work builds on IMEC’s iSiPP50G platform, which can be considered representative for advanced standardized silicon photonics, and further details on the platform have been published previously^52^.

**Fig. 2:**
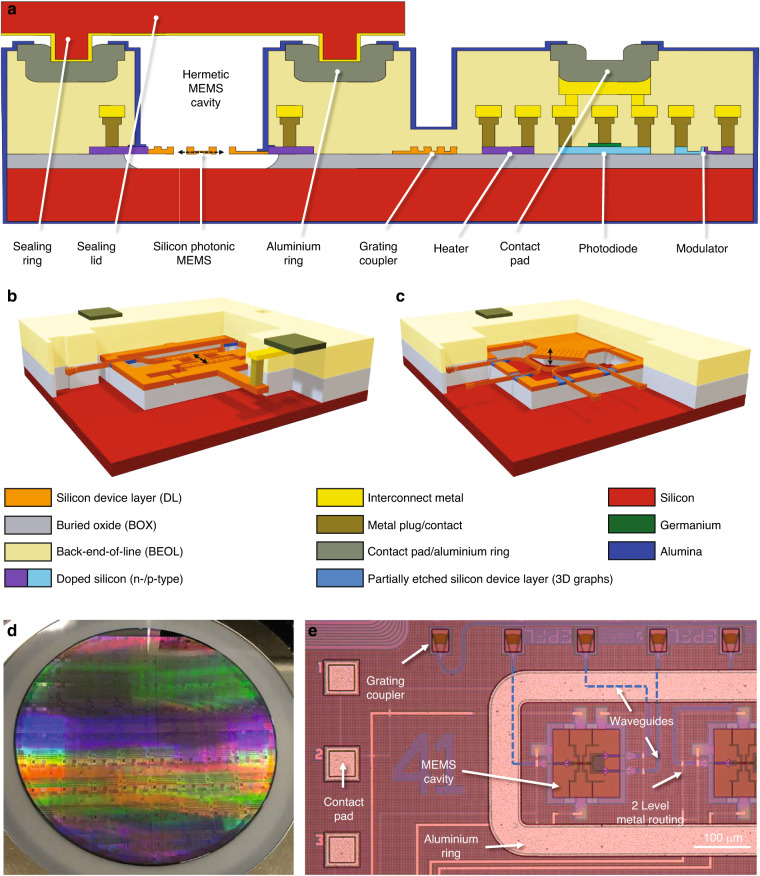
Integrated silicon photonic MEMS technology platform. The platform is based on IMEC’s iSiPP50G platform^52^, enhanced with custom postprocessing to produce suspended and movable MEMS structures alongside the platform’s standard components and with sealing caps above the MEMS structures to provide protection of the movable MEMS devices. **a** Schematic cross section of the technology platform and 3D perspective views of **b** in-plane and **c** out-of-plane movable devices to visualize conceptually the electrostatic actuators, the optical and electrical routing, as well as grating coupler and metal contact pads, serving as interfaces. **d** Photograph of a 200 mm silicon photonics wafer before MEMS release postprocessing and **e** microscope recording of a representative MEMS device integrated alongside high-performance grating couplers, low-loss waveguides for optical routing, and two metal levels for electrical routing. The final metallization layer serves as a bond pad for wirebonding, as well as an aluminium ring for the wafer-level hermetic sealing process

To introduce MEMS into standardized silicon photonics, the waveguides must first be made movable, and the MEMS actuators must second be implemented. Finally, to protect MEMS structures from environmental influences and for handling without the risk of damage, we have developed a wafer-level sealing process that allows placement of thin caps above the MEMS cavities. A simplified schematic cross-section of our silicon photonic MEMS technology platform is shown in Fig. 2a.

In principle, a variety of MEMS actuation methods, such as electrothermal, electromagnetic, or piezo-electric^53^ actuation, can be implemented in photonics^51^. Here, we choose electrostatic actuation due to the relative ease of process integration and lower power consumption compared to electrothermo-mechanical actuators. In particular, electrostatic actuators can be readily implemented using doped silicon as actuator electrodes, while electromagnetic or piezoelectric actuators require the integration of additional magnetic or piezoelectric materials, which are currently not available in standardized integrated photonics. Accordingly, we have developed a dedicated postprocessing procedure applied to the foundry processed silicon photonic integrated circuits that removes the sacrificial buried oxide selectively in the MEMS areas, as detailed in the methods section. We have implemented electrostatic actuators leveraging silicon device layer doping and electrical interconnects by metal routing layers to bond pads. Using a bias voltage between the handle layer and the device layer, an out-of-plane movement is achieved, while applying a voltage between two electrodes in the device layer induces in-plane displacement. The concepts for both in-plane and out-of-plane MEMS actuation in silicon photonics are shown schematically in Fig. 2b, c, respectively.

We fabricated our photonic MEMS design using the standard iSiPP50G platform that is processed on full 200 mm wafers, as shown in Fig. 2d. Our MEMS components are integrated alongside any of the high-performance library components available in the process design kit (PDK). A representative photonic MEMS device is shown in the microscope image of Fig. 2e, alongside several platform components, such as high-performance grating couplers, waveguide routing, two-level metal routing for electrical interconnects, and final metallization that is used for both the external electrical interfaces (bondpads) and for the wafer-level hermetic sealing process. To demonstrate the capabilities of our silicon photonic MEMS platform, we present a selected set of representative, experimentally demonstrated key functionalities exploring physical displacements at the microscale and nanoscale: optical power distribution and switching, phase shifting, and wavelength selective operations.

## Results

### Tunable power couplers and switches

**Fig. 3:**
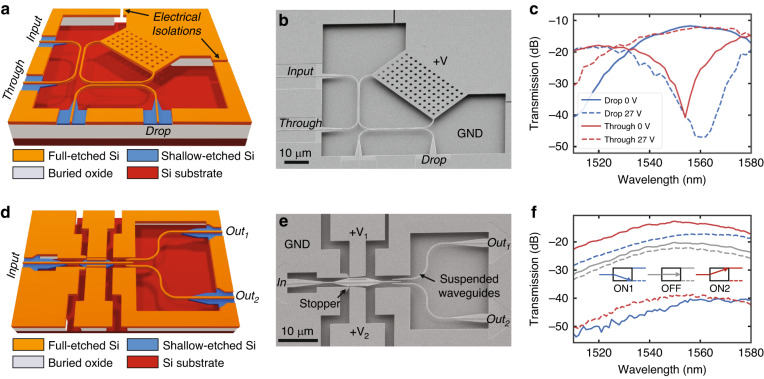
Tunable power couplers and switches. **a** 3-D schematic view of an out-of-plane actuated tunable power coupler indicating the input, through, and drop ports and the actuator. **b** SEM recording of the released device showing a cleanly suspended and initially well-aligned configuration of the couplers in the unactuated state. **c** Measurement results for the spectral characteristics of the tunable power coupler under DC actuation showing a maximum extinction ratio of 25 dB between the drop and through ports in the unactuated (0 V) state and a maximum extinction ratio of 30 dB in the actuated (27 V) state. The associated 3-dB bandwidth of this device is >30 nm. **d** 3-D schematic view of an in-plane actuated photonic 1 × 2 switch with the movable input and two fixed outputs. **e** SEM recording of the released device showing the curved electrodes, stoppers, and suspended waveguides. **f** Measurement results for the spectral characteristics of the device in all three functional states: ON1, OFF, and ON2, wherein the extinction ratio is >23 dB for both ON states and the bandwidth is >70 nm^59^

Power distribution and switching in PICs is commonly implemented by means of directional couplers consisting of two closely spaced waveguides undergoing evanescent field interactions^54^. For a given waveguide design and operating wavelength, the waveguide separation and interaction length will determine the amount of power coupled from the input port to either of the two output ports. For a conventional directional coupler, the output power ratio specified in the design phase is fixed after fabrication. With a MEMS-based directional coupler, the gap separation can be made variable, either vertically (out-of-plane actuation) or laterally (in-plane actuation), to allow redirection of the optical power from the input to either output. Here, we show the representative operation of a vertically actuated tunable power coupler. The power coupler consists of two suspended directional couplers that are attached orthogonally to the freestanding capacitor plate of an electrostatic MEMS actuator while keeping one branch of each directional coupler pair fixed, as schematically indicated in Fig. 3a. The directional couplers are based on our previously published design^55^; they have a coupling length of 30 µm, the initial lateral gap separation between the waveguides in the coupling region is 150 nm, and they are well aligned vertically upon release. A full transfer of optical power at a given operating wavelength between the two coupler branches can be achieved with an out-of-plane displacement of 300 nm. Additionally, we used waveguide tapering to obtain a particularly compact (20 µm × 30 µm) and spectrally broadband (>35 nm) means of optical power transfer. The insertion loss associated with this device is a modest 0.5 dB at the designed central wavelength of *λ* = 1550 nm and offers an extinction ratio of approximately 25 dB between the two fully switched configurations.

The adjustment of the vertical gap is achieved using an electrostatic actuator formed between a freestanding capacitor plate and the handle layer. By applying a voltage to the suspended silicon layer and keeping the substrate grounded, the suspended and free-moving arms of the directional couplers are pulled downwards by the generated electrostatic force. This configuration using out-of-plane movement requires an actuation voltage applied to a movable waveguide section. When considering the integration of multiple vertically actuated devices in larger circuits, the individual movable waveguide sections need to be isolated from each other to prevent short circuits or actuation of other devices in a circuit. Consequently, the tunable coupler device design is based on two sequential directional couplers with their respective movable branches attached to the MEMS actuation pad, which is isolated electrically by trenches through the device layer, as indicated by arrows in Fig. 3a. MEMS actuation increases the vertical gap separation between the movable and fixed arms of the coupler, thereby effectively decreasing the optical power coupling. Based on this operating principle, in the unactuated state, light injected at the input port shown in Fig. 3a couples completely over the first directional coupler and then again in the second coupler, exiting at the drop port. When the actuator is engaged such that it has been displaced approximately 300 nm down towards the substrate, there is no coupling in the first directional coupler, and all the optical power exits the device at the through port.

Figure 3b shows a fabricated tunable coupler after the MEMS release postprocessing step. The footprint of the device is 78 µm × 78 µm. The release process results in a well-released device without visible residues and with both directional coupler pairs well aligned, which indicates low residual stress in the suspended waveguides. Optical characterization results are shown in Fig. 3c. In the idle, nonactuated state (0 V actuation voltage), the optical power is sequentially coupled from the input port through the two directional couplers to the drop port. At the operation wavelength, an extinction ratio of 25 dB is achieved between the drop and the through port. In the actuated state (27 V), optical power is concentrated in the drop port, with an extinction ratio of 30 dB with respect to the drop port. The 3 dB spectral bandwidth of the device is >30 nm. The actuation voltage for vertical actuators can be reduced by using a softer suspension design, with the trade-off of increased footprint and increased response time, and we have previously demonstrated vertically actuated silicon photonic MEMS switching with an actuation voltage as low as 3.75 V^56^.

In addition to out-of-plane actuated tunable couplers, our platform also allows for the implementation of in-plane actuated devices. For example, the directional coupler described above can be attached to an in-plane electrostatic comb-drive actuator, and from an initial 150 nm lateral gap, an in-plane displacement of a mere 50 nm is sufficient to achieve full power coupling^57^.

To demonstrate the versatility of the platform, we summarize our results for an in-plane actuated broadband and compact, single-pole double-throw switch, as schematically illustrated in Fig. 3d. The device consists of a single suspended-free input waveguide that is initially centred between the two suspended-fixed output waveguides so that only symmetric power transfer occurs (IDLE state). When an actuation voltage is applied to actuation electrode 1 or 2, an electrostatic force pulls the grounded input waveguide in-plane towards the selected actuation electrode, therein aligning it to the waveguide associated with Output 1 or Output 2, respectively. For example, with electrode 1 engaged, the input waveguide is aligned to the Output 1 waveguide, which receives the complete injected optical power (ON1 state). By applying the actuation voltage to electrode 2, the scenario is flipped, and Output 2 receives all the optical power (ON2 state). We choose to operate a curved electrode electrostatic actuator design^58^ in the pull-in regime, wherein the electrostatic force exceeds the restoring mechanical spring force and the input waveguide snaps towards the electrode and corresponding output waveguide. This allows for switching of optical power in a 1 × 2 configuration. To mitigate the risk of the input waveguide pushing against the output waveguide upon actuation, a pair of mechanical stoppers are also included in the design. These structures are presented in Fig. 3e, which shows the device after successful MEMS postprocessing and release. Further highlighted are the curved electrodes, which offer a better force versus footprint trade-off than their flat counterparts and consequently allow for a more compact actuator design.

Characterization of this device was performed similar to that of the out-of-plane variant. A wavelength sweep where light is injected into the Input and then measured at Output 1 and Output 2 is performed in the unactuated state to capture the IDLE state (Fig. 3f). Subsequently, an actuation voltage is applied in sequentially increasing steps to either electrode 1 or electrode 2, and the change in relative power transmission to both outputs is recorded. The results of these measurements are summarized in Fig. 3f, which depicts the optical characteristics from 1510 to 1580 nm for the unactuated (0 V) and actuated (22 V) ON1 and ON2 states. An extinction ratio of approximately 23 dB is maintained over the entire 70 nm wavelength sweep, which is indicative of particularly broadband behaviour. A step-response measurement reveals a response time of 0.73 µs when switching from IDLE to ON1 and 0.82 µs from the ON1 to IDLE state. Furthermore, the device’s 65 µm × 62 µm form factor is particularly small, making it an attractive component for large-scale PICs. Additional details on the design, simulation, and characterization can be found in ref. ^59^. The reported measurements of the switches in Fig. 3c, f include the grating couplers and on-chip propagation losses, and dedicated measurements were performed to estimate the device insertion losses. The experimentally extracted insertion loss for individual both in-plane and vertically actuated switches was <0.5 dB at 1550 nm. FDTD simulations predict 0.02 dB and 0.24 dB at 1550 nm for the vertically moving and in-plane actuated switch, respectively. The experimentally observed losses are upper bounds, as they also include losses originating from the transitions to the MEMS cavity (cf. section ‘Optical crossings and transitions’) and from tolerances in alignment and fabrication.

### Tunable phase shifter

Silicon photonic circuits often require control over the phase in addition to the power. For instance, beam-steering circuits require precise control over the phase differences between waveguides reaching an array of antennas. Moreover, phase shifters can be used in interferometric circuits, such as MZIs, to tune power. A common method to introduce a phase shift in a waveguide is to tune its effective index, according to Eq. 1.

1 $${\Delta}\phi = 2\pi \frac{{L{\Delta}n_{{\rm{eff}}}}}{\lambda }$$

where Δ*ϕ* is the phase shift, *L* is the waveguide length, *Δn*_eff_ is the change in the effective index, and *λ* is the wavelength.

**Fig. 4:**
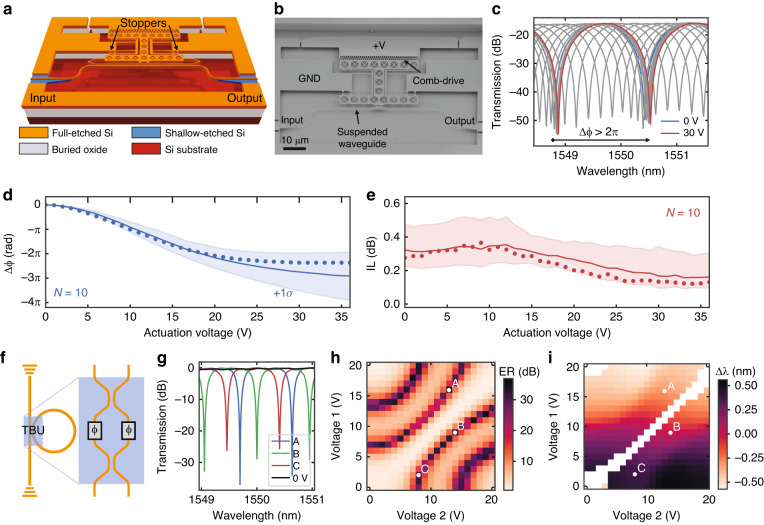
Tunable phase shifter. **a** 3-D schematic view of an in-plane actuated phase shifter. **b** SEM recording of the phase shifter, comprising a suspended waveguide and a comb-drive actuator to tune the modal cross-section of the waveguide by pulling away a 220 nm wide silicon beam attached to the movable shuttle^60^. **c** Characterization of the phase shifter within an unbalanced Mach Zehnder interferometer (MZI), displaying an over 2*π* phase shift and low insertion loss (large extinction ratio). **d** Extracted phase shift and **e** insertion loss versus voltage at a wavelength of 1550 nm. Dotted: single device, line: median for *N* = 10 devices, shaded region: +/−1*σ*. **f** Tunable basic unit composed of two MEMS phase shifters, one per arm of a suspended balanced MZI, here configured as an all-pass ring resonator. **g** Example characterization of the tunable basic unit with two MEMS phase shifters, showing versatile control over the ring filter response and a free spectral range of 0.93 nm at a wavelength of 1550 nm. **h** Actuation map of the corresponding filter extinction ratio and **i** resonance tuning at a wavelength of 1550 nm. For low values of the extinction ratio, no resonances were observed, and no resonance shift was extracted

State-of-the-art programmable photonic circuits rely on thermal tuning of the effective index, but heaters are power-hungry, typically consuming over 1 mW per device to effect a π phase shift. MEMS devices based on electrostatic actuators display negligible power consumption (<1 μW) and can provide large effective index tuning due to the modification of the waveguide cross-section. We have demonstrated such phase shifters on our silicon photonic MEMS platform, reaching (2.9*π* ± *π*) at a wavelength of 1550 nm, with a low insertion loss of $0.33_{ - 0.10}^{ + 0.15}$ dB^60^. Our phase shifter relies on a comb-drive actuator for in-plane modification of the waveguide cross-section (see Fig. 4a). As the bias is applied between the fixed and movable comb-drive teeth, the movable shuttle pulls a 220 nm wide silicon tuning beam away from the 375 nm waveguide core and decreases the optical index. The phase shift is achieved within a short waveguide length of 50 μm for an overall device footprint of 100 × 45 μm^2^ (Fig. 4b).

We characterized the phase shifter by inserting it into one arm of a length-unbalanced MZI. Interference fringes are visible at the output of the MZI (Fig. 4c). We obtained both phase shift (Fig. 4d) and device insertion loss (Fig. 4e) with respect to voltage and wavelength for *N* = 10 devices by fitting the spectra to an MZI model. Finally, the device greatly benefits from the selective doping of the device layer provided by the iSiPP50G platform. We measured an *f*_*−*3 dB_ bandwidth of 1.03 MHz in air, which was not limited by the electrical cut-off, unlike reported MEMS actuators on standard SOI platforms.

In programmable photonic circuits, phase shifters are used within balanced MZIs to form tunable basic units (TBUs), corresponding to 2 × 2 analogue blocks with full control over both power and phase. We demonstrated such a TBU based on our previously published phase shifter using a suspended MZI and two phase shifters in a compact MEMS cavity (see Fig. 4f). We tested the TBU as part of an all-pass ring resonator and measured the transmission spectra from 0 to 20 V for both phase shifters; example spectra are shown in Fig. 4g. The resulting filter exhibits a tunable extinction ratio from 0 to 36 dB (Fig. 4h), and its frequency can be tuned beyond the free spectral range (FSR) of the ring (Fig. 4i).

### Tunable add-drop filter

In addition to controlling the amplitude and phase of light propagating in a PIC with tunable power couplers and phase shifters, MEMS can selectively manipulate individual wavelengths. For example, in a wavelength division multiplexed (WDM) system, each optical carrier is assigned a unique wavelength, which allows increased system-level bandwidth. Adding (sending) or removing (receiving) data to/from one of these communication channels requires a wavelength-specific filter, and an efficient implementation for precise and efficient filtering is the ring resonator. Consequently, we present a tunable add-drop filter that can be engaged or disengaged through the use of MEMS-based vertical displacements^61^.

**Fig. 5:**
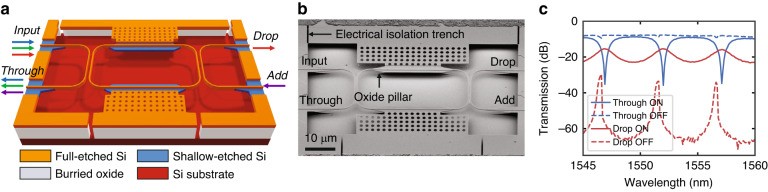
Tunable add-drop filter. **a** 3-D schematic view of the out-of-plane actuated tunable add-drop filter depicting the resonant-mode behaviour of the engaged filter. **b** SEM recording of the released device showing the suspended ring and the two well-aligned and suspended bus waveguides on either side. **c** Measurement results for power transmission to the through and drop ports for both on- and off-resonance wavelengths in the two filter states. When the filter is ON (0 V), the free spectral range (FSR) is ~5 nm, and the port extinction ratio (ER) is 20.4 dB for resonant wavelengths and 13.2 dB for off-resonance wavelengths. Turning OFF the filter (27 V) maintains an ER > 20 dB for resonant wavelengths

Due to its resonant nature, the spectral response produced by a ring resonator is particularly narrow-band and steep, allowing filter designs with a high extinction ratio. This high performance is made possible because the optical path length of one of the ring’s supported resonant modes is carefully designed to coincide with an integer multiple of the operation wavelength. The design and operating principle of our MEMS-actuated tunable add-drop filter is schematically illustrated in Fig. 5a and Fig. 5b shows a scanning electron microscope (SEM) image of a fabricated device. The ring resonator structure is flanked by a pair of suspended bus waveguides for coupling/decoupling light and is also attached on two sides by parallel-plate capacitor actuators. In this initial configuration, where the ring is well aligned in-plane to the two bus waveguides, the filtering function of the device is engaged (ON). Here, resonant wavelengths injected at the input port in the first bus waveguide couple to the ring and propagate until they couple out to the second bus waveguide and exit via the drop port. Concurrently, light of off-resonance wavelengths continues along the first bus waveguide and exits the through port. During the “Add” operation, light is injected at the Add port, coupled to the ring, and then coupled over to the first bus waveguide to combine with any other light passing to the Through port. The filter is disengaged, i.e., turned OFF, when an actuation voltage applied to the suspended ring pulls it down and out of alignment with both bus waveguides towards the grounded substrate. In this state, light from the input port simply passes to the through port without wavelength selectivity.

Characterization results for the device in the ON and OFF states are provided in Fig. 5c, which depicts the optical power transmission to the through and drop ports in a narrow wavelength sweep from 1545 nm to 1560 nm. With the filter engaged (ON), there is a clear dip in the optical power transmitted to the through port at resonant wavelengths, and complementarily, there is a peak in transmission for these wavelengths at the drop port. Quantitatively, these characteristics correspond to port extinction ratios (ER) of 20.4 dB for resonant wavelengths (e.g., *λ* = 1552 nm) and 13.2 dB for off-resonance wavelengths (e.g., *λ* = 1549 nm). The measured 5 nm between consecutive minima and maxima in the through and drop ports, respectively, is the free spectral range (FSR) and matches closely with calculated and simulated values. In the coupled state, we measure a resonance line width of 1 nm, corresponding to a quality factor of 1552 and a bandwidth of 124 GHz. After electrostatic MEMS actuation at 27 V, the ring is displaced approximately 500 nm downwards to the substrate, thereby bringing it out of alignment with both bus waveguides and disengaging the filter (OFF). The linewidth decreases to 0.26 nm, and the quality factor converges to 5969 (bandwidth of 32.4 GHz) when the coupler is fully disengaged. This value is representative of the intrinsic losses in the ring. The rather flat optical response in the through port indicates that in this configuration, the device exhibits little wavelength specificity and acts more as a bus waveguide than as a wavelength filter. Off resonance, the isolation of the drop port exceeds 50 dB; however, a residual amount of optical power couples to the ring at the resonance wavelengths and thus to the drop port, as indicated by the peaks in the drop port transmission in Fig. 5c, maintaining a port extinction of >20 dB. Because the device utilizes a ring resonator, it can be compact (45 µm × 75 µm); when combined with its strong optical performance, this tunable add-drop filter presents as a suitable candidate for integration in a larger-scale PIC-based WDM solution.

### Optical crossings and transitions

**Fig. 6:**
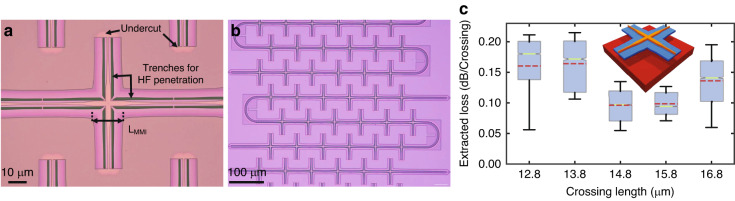
Optical crossings. **a** Microscope image of a multimode interference (MMI)-based optical waveguide crossing. MMI length (L_MMI_), trenches along the MMIs for penetration of hydrofluoric acid (HF) and the undercut regions are annotated. **b** Microscope image showing cascaded crossings to determine the insertion loss of the MMI-based waveguide crossings. **c** Plot showing the spread of the experimentally extracted insertion loss values over the measured wavelength range for different MMI lengths used for the crossing. The box extends from the lower to upper quartile values, the red dotted line represents the mean, and the light green line indicates the median. Inset shows the 3-D drawing

In contrast to electronic integrated circuits, where multiple metal layers are available for electrical routing, currently available integrated silicon photonics platforms based on silicon-on-insulator (SOI) technology typically offer only a single waveguide layer for optical routing. Consequently, to realize complex circuits with high integration density of functional blocks, low-loss waveguide crossings with minimal crosstalk are needed. Multimode interference (MMI)-based waveguide crossings are usually used for this purpose, as low loss can be achieved by designing the optical mode propagating to self-image at the centre of the waveguide intersection^62^. Such low-loss MMI crossings are already available as a building block in IMEC’s standard silicon photonics platform iSiPP50G^26^. However, the performance of the standard waveguide crossings is not optimized for use within the MEMS cavities since the back-end-of-line (BEOL) stack above and the buried oxide (BOx) underneath the waveguides have been removed to facilitate the mechanical movements (Fig. 5a). This removal of the oxide around the waveguides changes the index contrast, and the design parameters for the MMIs then need to be adjusted. To achieve the self-imaging condition of the mode right at the intersection, we swept the length of the MMIs while keeping the width constant. To experimentally extract the insertion loss, we use specifically designed loss test structures with various counts of sequential crossings, as shown in Fig. 6b. We experimentally demonstrate an insertion loss of <0.1 dB per crossing for an MMI length of 14.8 µm (Fig. 5c; further details can be found in the ‘Materials and methods’ section).

**Fig. 7:**
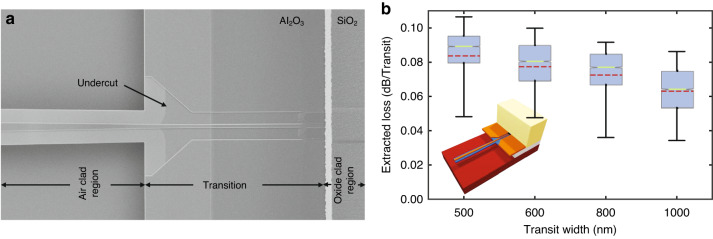
Optical transitions. **a** Scanning electron microscope (SEM) image of an optical transition from the oxide clad region to the cavity where the back end of line stack and the buried oxide have been removed using the developed wafer level postprocessing on the standard silicon photonic platform (iSiPP50G) chip. **b** Plot showing the spread of the experimentally extracted insertion loss values over the measured wavelength range for different core widths of the shallow etch waveguide-based optical transitions. The box extends from the lower to upper quartile values, the red dotted line represents the mean, and the light green line indicates the median. The inset shows a 3-d representation of the optimized optical transition

Since both BEOL and BOx in the MEMS cavity region are removed to make the waveguides free-standing, the optical waveguides also need to pass the interface between the BEOL stack and the MEMS cavity. Due to the sudden change in refractive index (around the waveguides) at the interface, back-reflections within the waveguides occur. To suppress these back reflections and the resulting additional losses, we designed shallow-etch waveguide-based optical transitions. Shallow-etch waveguides are used because the fundamental optical mode exhibits stronger confinement within the silicon and thus lower interaction with the cladding. This decrease in interaction with the cladding minimizes the effect of the sudden change in refractive index and minimizes reflections and associated losses. The scanning electron microscope image in Fig. 7a shows a representative fabricated transition. We swept the width of the shallow etch waveguide core to determine the optimized transitions with the least insertion loss. Similar to those for optical crossings, optical paths with different numbers of transitions were designed to experimentally characterize the insertion loss of these optical transitions. The measurement results in Fig. 7b show that transitions with wider waveguide cores experience smaller insertion losses. This measurement is in agreement with expectations, as the interaction of the optical waveguide mode decreases with increasing waveguide core width.

### Wafer-level hermetic sealing of the silicon photonic MEMS

Nearly all silicon photonic MEMS devices feature suspended and movable parts, including the devices presented above. To ensure reliable operation and protect suspended silicon photonic MEMS devices from environmental influences, such as dust and varying humidity levels, the devices must be placed inside hermetically sealed cavities^63^. Therefore, we have developed a platform for wafer-level hermetic sealing of silicon photonic MEMS by wafer bonding^64^. This approach is a wafer-scale process utilizing commercial semiconductor wafer bonding equipment. It is fully compatible with the PIC silicon photonic foundry platform (iSiPP50G, IMEC) and features optical and electrical feedthroughs from the inside to the outside of the hermetically sealed cavities containing the silicon photonic MEMS.

**Fig. 8:**
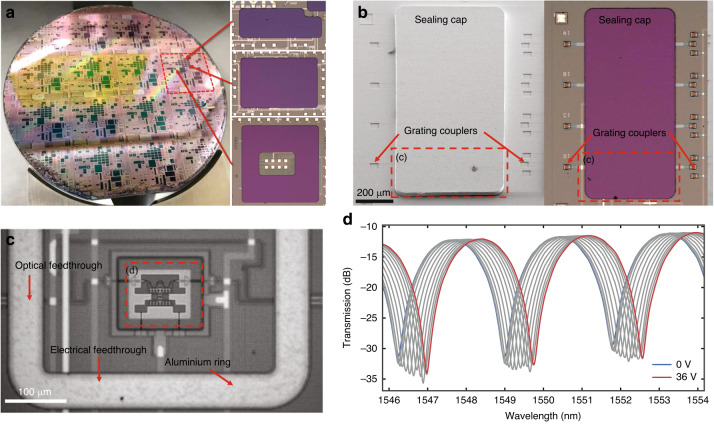
Wafer-level hermetic sealing. **a** Hermetically sealed silicon photonic MEMS devices on a 100 mm diameter wafer. The hermetic sealing approach uses wafer bonding for cap transfer and enables versatile shapes and designs of the sealed cavities with surrounding bond pads. **b** Top SEM and optical microscope images of the transferred caps on top of the hermetically sealed cavities. **c** Top microscope view of a MEMS phase shifter before wafer bonding and sealing, enclosed by the aluminium ring against which the cap will be bonded. **d** Successful transmission measurement of a MEMS phase shifter (see section ‘Tunable phase shifter’ above) after sealing and dicing, using an actuation voltage of up to 36 V^64^

In our sealing platform, we used 25 µm-thick silicon sealing caps that are transferred from a handle wafer onto the cavities containing the silicon photonic MEMS structures and hermetically seals them through wafer bonding. The preprocessed cavities with the silicon photonic MEMS are surrounded by a 20 µm-wide protruding aluminium ring (Fig. 2a). The corresponding rings of the sealing caps that are placed on a separate handle wafer consist of a 2.2 µm-thick stack of tungsten-titanium/gold. For cap transfer, we used metal-to-metal thermocompression wafer bonding at a temperature of 250 °C (see ‘Materials and Methods’ section for more detail). The demonstration of successfully sealed silicon photonic MEMS on a 100 mm diameter wafer is shown in Fig. 8a. The shown wafer included 672 individual cavities that were hermetically sealed by sealing caps, featuring a vacuum sealing yield of 90% of the cavities. The shape of the cavities and caps can be flexibly designed to fit the device and system needs (insets in Fig. 7a), with currently demonstrated cap dimensions ranging from 450 µm × 330 µm to 2800 µm × 2950 µm.

Vacuum-sealed PICs require optical and electrical feedthroughs to connect the silicon photonic MEMS devices inside the sealed cavities to the outside components. In our sealing platform, the optical and electrical feedthroughs from the inside of the cavities to the outside are buried within the BEOL stack and travel underneath the hermetic sealing rings. To electrically and optically interface the sealed photonic MEMS, both the input and output (I/O) grating couplers and the electrical bond pads are located at the wafer surface outside the sealed cavity, surrounding the cap (Fig. 8b).

We demonstrated the compatibility of our sealing platform with silicon photonic MEMS by hermetically sealing photonic MEMS phase shifters in cavities containing vacuum. We used an MZI and optical and electrical feedthroughs to interface and evaluate the sealed MEMS phase shifters (Fig. 8c). The phase shifter shown here has a curved waveguide in the phase shifting section that provides better phase shift linearity with applied voltage, at the cost of reduced maximum phase shift, compared to the device in Fig. 3. We measured the device transmission at a wavelength of approximately 1550 nm for different actuation voltages, confirming that the suspended MEMS structures were suspended and could be actuated. These measurements also confirmed that the electrical and optical feedthroughs were not negatively affected by the sealing process. Moreover, after sealing the MEMS phase shifter, we observed an increase in the mechanical quality factor for the fundamental mode of the comb-drive actuator. The mechanical Q of the device increased from 8 to 36 after sealing. A reduction in air damping due to the presence of a vacuum inside of the cavity can explain this data. The results conclusively demonstrate that the photonic MEMS phase shifters were hermetically sealed in a vacuum cavity and operated as intended.

### Electronic–photonic assembly with scalable electrical and optical interfaces

**Fig. 9:**
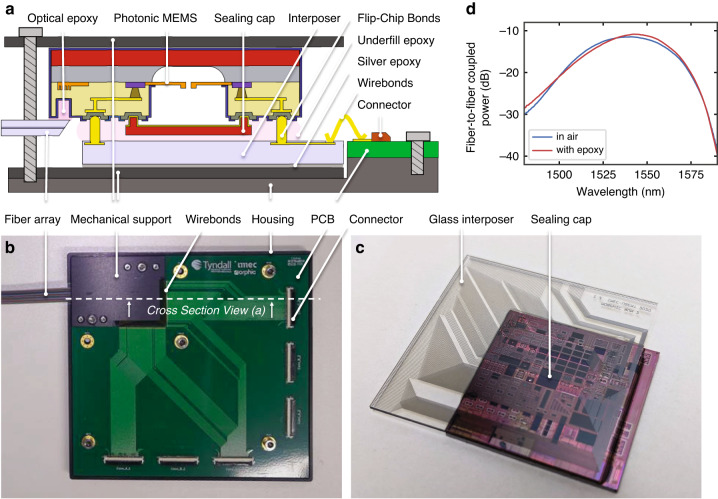
Electronic-photonic assembly. **a** Schematic cross-section representation and **b** photograph of the electronic-photonic assembly. **c** Subassembly of the silicon photonic MEMS die flip-chip bonded to the glass interposer. The assembly consists of the interposer-chip subassembly, attached to a mechanical support by silver epoxy and wirebonded to the printed circuit board with fan-out to high-density connectors, a 72-channel fibre-array attached by index matched epoxy to the PIC, and a metal housing to support the chip-interposer subassembly. **d** Using active alignment with shunt connectors on the chip allows for low insertion loss fibre-to-chip coupling over the telecom C-band

To leverage large-scale silicon photonic MEMS circuits, high counts of electrical (>10^3^) and optical (>50) in- and output interfaces must be integrated. In our technology platform, we make use of very thin sealing caps (<30 μm), as shown in the schematic cross-section representation of the electronic–photonic assembly in Fig. 9a. A photograph of the full assembly is depicted in Fig. 9b. The low sealing cap height allows us to use bond stud bumps on the silicon photonic MEMS chip and flip-chip bond to a matching pattern with solder-jetted bond bumps on a glass interposer (Fig. 9c). The glass interposer features a single passivated metal redistribution layer, with openings solely over the bond pads and wirebond pads, to route the electrical interface to the edge of the interposer, which makes it compatible with wire-bonding to printed circuit boards (PCBs). Here, we show wirebonding to redistribution PCBs connected to high-density flex connectors, yet the assembly approach is also compatible with any customized PCB. By design, the glass interposer does not cover the silicon area entirely to allow for epoxy-attach of quasiplanar fibre-arrays. Here, we integrate a 1 × 72 fibre array to a matching 1 × 72 grating coupler array on-chip. We use active alignment of shunt waveguides, resulting in fibre-to-chip coupling with a minimum of 10.4 dB fibre-to-fibre coupling loss after assembly, including epoxy (<15 dB over the entire C-band due to the spectral response of the grating couplers, as shown in Fig. 8c). Further details on the assembly process are described in the ‘Materials and methods’ section.

## Discussion

We have demonstrated fully wafer-compatible integration and hermetic sealing of MEMS in IMEC’s iSiPP50G silicon photonics foundry process. The MEMS release process seamlessly follows the standard silicon photonics processing sequence and is fully compatible with the already developed high-performance library components offered by the foundry. We thus enhance standard silicon photonics capabilities with MEMS components, and we have developed dedicated low-loss crossings and transitions from and to photonic MEMS devices. Our hermetic sealing process at the wafer scale displays small and flexible footprints and is compatible with large numbers of optical and electrical inputs and outputs. Our demonstrated fundamental building blocks showcase the benefits of MEMS integration in silicon photonics with fast and precise actuation of up to several hundred nanometres displacement with low power consumption. While the MEMS release process and the hermetic sealing process are currently performed as postprocessing steps in university clean rooms, they are entirely compatible with 200 mm wafer scale fabrication and could be integrated as a standard or optional process module in the foundry process flow (with sufficient customer demand). In addition, the low sealing cap height offers compatibility with subsequent flip-chip bonding to electrical interposers with high counts of electrical in- and outputs for high-density packaging of photonic integrated circuits while allowing integration of high counts of optical inputs and outputs by interfacing linear arrays of grating couplers to quasiplanar fibre arrays. Our current solution can already enable on the order of 10^3^ electrical interfaces and on the order of 10^2^ optical interfaces. The electrical interfaces can be scaled further by direct flip-chip bonding of electronic integrated circuits, and optical interfaces can be scaled further, for example, by multicore optical fibres or photonic wirebonding, areas of research that are currently being investigated.

Our comprehensive experimental report of selected devices demonstrates that silicon photonic MEMS is a promising technology basis that is capable of rapidly maturing. The performance of our demonstrated silicon photonic MEMS devices is summarized in Table 1 and compared to representative devices based on the most commonly implemented physical effects for reconfiguration in photonic integrated circuits. The summary table underlines that silicon photonic MEMS devices are uniquely suited for large-scale integration, as they provide an unrivalled combination of simultaneously low loss, low power consumption and broadband performance with a compact footprint. While high-speed modulators are optimized for even faster operation, silicon photonic MEMS exhibit response times <1 μs, which enable fast photonic circuit reconfiguration exceeding MHz speeds.

**Table 1:** Silicon photonic MEMS device performance in comparison with state-of-the art free carrier dispersion (FCD), thermo-optic (TO), plasmonic-organic hybrid (POH), lithium niobate (LiNbO_3_) and liquid crystal (LC) devices, where silicon photonic MEMS uniquely provide a compact footprint, low insertion loss, low power consumption and broad optical bandwidth

	Property/performance					
Photonic device	Footprint (incl. cont.) [μm × μm]	Insertion loss [dB]	Drive voltage [V]	Power cons. [nW]	Optical bandwidth [nm]	Response time [μs]
MEMS tunable coupler	78 × 78	0.5	27	–^a^	30	–^a^
MEMS switch	65 × 62	–^a^	23	–^a^	1500–1600	<1
MEMS ring resonator	75 × 45	–^a^	27	–^a^	0.26	–^a^
MEMS phase shifter	100 × 45	0.33	20	<1^b^	50^f^	2^c^
TO phase shifter^36^	109 × 21	1.23	0.8	2560	1520–1600	<34.8
FCD MZI phase shifter^68^	2100 × 2000	6.9	0.5	203·10^3^	Broadband	<0.001
POH phase shifter^39^	29 × 10	12	3	2.4·10^6d^	Broadband	<0.001
LiNbO_3_ phase shifter^69^	20,000 × 500	0.5	0.37	26·10^3e^	Broadband	<0.001
LC phase shifter^70^	80 × 40	0.83	10	–	Broadband	1000

*MEMS* microelectromechanical systems, *FCD* free carrier dispersion, *MZI* Mach‒Zehnder interferometer, *TO* thermo-optic, *POH* plasmonic-organic hybrid

^a^Conclusive experimental data not reported due to limited availability of dedicated test structures

^b^Measured dynamic *π* phase shift modulation at 1 kHz

^c^Measured mechanical resonance frequency

^d^Modulation at 40 Gb/s with 60 fJ/bit

^e^Modulation at 70 Gb/s with 0.37 fJ/bit

^f^Based on the optical bandwidth corresponding to a phase shift within [1.5*π*, 2.5*π*]

The optical losses can be optimized at the device level through individual component design and at the circuit level through placement strategies in hermetically capped subcircuits that minimize transition losses. Due to electrostatic actuation, power consumption for individual devices is typically on the order of nW^60,65^. While in principle, power consumption is only required for charging the capacitor, leak currents and power supplies do contribute to the system level power consumption in the steady state. Here, MEMS can provide an additional unique capability to exhibit no power consumption in the steady state by exploiting latching mechanisms. For example, bistable nonvolatile states have been implemented by adhesion forces in switches^66^, yet devices with multiple nonvolatile states can also be designed to enable digitally tunable couplers or phase shifters, developments for which are currently underway and can readily be implemented in our silicon photonic MEMS platform. Our demonstrated response times reach below 1 μs, limited by mechanical inertia. Our electrostatic MEMS actuators typically require driving voltages up to 30 V for maximum operation. Both response time and actuation voltages can be adjusted by appropriate MEMS design, and CMOS-compatible actuation voltages can also be obtained in our technology^56^. Decreasing the response time for a given actuator design requires a stiffer MEMS suspension design, which in turn increases the actuation voltage. Thus, in a given technology platform, a trade-off between fast response time or low actuation voltage has to be made.

The demonstrated devices represent examples of a versatile and powerful silicon photonics platform extension with MEMS. The potential of this technology extends far beyond our selected demonstrations. Namely, we predict that a myriad of devices can be implemented in standardized silicon photonic MEMS, including microphones, optomechanical sensors, spectrometers, accelerometers, pressure sensors, Brillouin scattering-based sensors, etc. In particular, since the electrostatic actuation mechanism lends itself to cryogenic operation, we speculate that silicon photonic MEMS can be an excellent candidate for integrated quantum photonics. With our integration of silicon photonic MEMS in standard silicon photonics, we also ensure both backwards- and forwards-compatibility with future enhancements of the silicon photonics platform, such as heterogeneous integration of laser sources or lithium niobate modulators, the combination of which can provide pathways towards MEMS tunable lasers for spectroscopy.

This unique combination fulfils the requirements of scalability and provides outstanding potential for the integration of MEMS in very large-scale photonic integrated circuits. For example, our phase shifters and tunable couplers constitute an elementary basis for integrated linear optics, and our switches and tunable filters underline the potential for applications in telecommunications, for example, in wavelength-selective switches. Owing to the unique scalability, silicon photonic MEMS thus provide unprecedented potential compared to other photonic integrated technologies for emerging applications, such as photonic accelerated computing, 3D imaging for autonomous vehicles, or quantum information processing.

## Materials and methods

### Design methodology, mask layout and fabrication

We use finite element modelling and a finite difference time domain solver for the MEMS and photonic design, respectively. The optimized designs of individual devices are enclosed by a silicon rim design delimiting the MEMS cavities. The rim includes doped silicon regions that provide localized high conductivity for applying voltages to the MEMS actuators, as well as electrical isolation trenches to separate regions at different potentials. The rim also contains optical transits, whose carefully designed partial-etched silicon geometries enable a low-loss transition from the BEOL stack, oxide-clad region, to the MEMS cavity, air-clad region. These transitions also reduce the effect of back reflections. At the other interface of the silicon rim, within the BEOL stack, are the waveguides carrying light to the input and output grating couplers and the metallization connected to the contact pads. An optional aluminium ring can be included around the MEMS cavity, which can be used in conjunction with a sealing cap bonded after the MEMS release process, providing additional encapsulation against environmental perturbations.

**Fig. 10:**
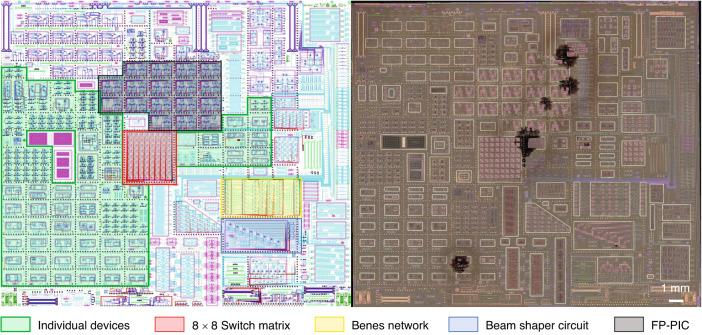
Silicon photonics chip design. Layout (left) of the full silicon photonics chip provided for fabrication at IMEC. Regions highlighted in green indicate the portions of the chip allocated to the testing of new geometries, functionalities, etc., at the individual device level. The red, yellow, blue, and black regions indicate the positions of four representative large-scale circuits utilizing a large number of individual devices. A microscope image (right) of the chip after fabrication and after custom MEMS postprocessing captures the full layout and scale. The aluminium rings are visible as bright contours, as is the exposed device layer silicon seen in pink inside the MEMS cavities. Note that the dark spots in the image are regions where the integrity of the alumina passivation was locally compromised and the vapour HF etchant could penetrate into the BEOL stack

Individual devices are arranged and placed on the chip for immediate testing in the lab following MEMS release. Larger-scale circuits consist of several such devices connected to one another. These circuits not only take up more real estate on the chip and must therefore be strategically placed but must also undergo additional electrical and optical packaging before testing because of the large number of contacts and inputs/outputs. Figure 10 shows both the layout and a microscope image of the full chip, indicating regions that have been allocated for individual device testing, as well as those reserved for particular multicomponent functions, e.g., switch matrices and beam shaping.

### MEMS release process

Fabrication of the samples is carried out at IMEC’s 200 mm wafer-scale facility, after which wafers are diced into individual 46 mm × 46 mm coupons each consisting of four 23 mm × 23 mm chips in a two-by-two arrangement. This coupon format is convenient for postprocessing in the equipment in the EPFL’s Center of MicroNanoTechnology (CMi), but it should be noted that all of the subsequently discussed process steps are also entirely compatible with 100 and 200 mm wafers.

#### Spin coating and lithography

**Fig. 11:**
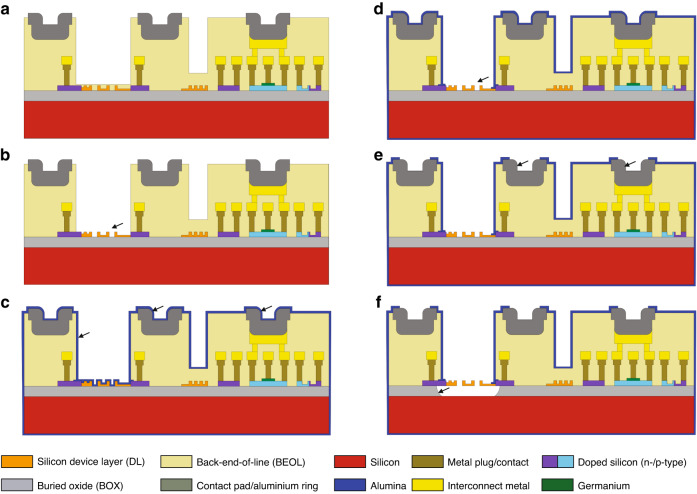
Simplified process flow indicating the main steps used in MEMS postprocessing. Step **a** shows the initial cross-section of the sample after processing at the foundry. The first oxide removal step **b** clears any remaining oxide on top of and between the waveguides before an alumina passivation layer is conformally deposited over the entire sample using atomic layer deposition (ALD) in step **c**; this layer serves as a hard mask against the vapour phase hydrofluoric acid (vHF) etchant used to selectively remove the sacrificial buried oxide (BOx) layer. Following passivation, the alumina must be opened up inside the MEMS cavities in step **d** to provide vHF access to the BOx, as well as over the metallization in step **e** to ensure proper electrical contact. In a final step **f**, vHF is used to remove the BOx under the exposed device layer silicon, resulting in free-standing, movable silicon photonic MEMS structures. Arrows serve as visual guides on the locations where the corresponding process step is applied

An initial cross-section of the unprocessed samples, as they arrive from the foundry, is provided in Fig. 11a. We make use of a standard foundry process module to create an opening in the back-end-of-line stack above the silicon device layer to provide access close to the waveguides, resulting in significant topography on the samples. While topography is also present in the area of the grating coupler windows and to a lower degree at the location of the contact pads, the step height of the back-end-of-line opening is the most important topographic feature and amounts to ~6.3 µm. This topography poses challenges for spin coating of photoresists and for exposure. For spin-coating, we use a 4.5 µm thick layer of AZ ECI 3027, a chemically amplified i-line positive photoresist, providing good step coverage of all topography features. The exposure was performed using a Maskless Aligner (Heidelberg Instruments MLA 150) at a 405 nm exposure wavelength. The use of the mask-less aligner allows for a rapid adjustment of the exposure features during process development, and the i-line laser-based exposure allows us to focus on topography features and circumvent the challenges of divergence in mask-based exposure. Exposure parameters for dose and focus are optimized in calibration routines before exposure. Development is performed manually in a TMAH-based developer solution (AZ 726 MIF). To remove any residual photoresist polymer after development, a descum oxygen plasma strip is performed (Tepla GiGAbatch, 200 W, 15 s). After patterning, the photoresist is removed in Microposit Remover 1165 at 80 °C followed by an oxygen plasma descum (Tepla GiGAbatch, 200 W, 3 min). This lithography sequence is used for all lithography steps in the postprocessing.

#### Oxide etch

After the foundry process, the volumes between waveguides are filled with silicon oxide, and in the area of the back-end-of-line windows, the waveguides are covered with a 5 nm thin oxide layer. To cleanly remove these oxides, we apply a 3 min liquid buffered hydrofluoric (BHF) acid etch (Fig. 11b).

#### Alumina passivation

We deposit 50 nm Al_2_O_3_ (alumina) by atomic layer deposition (BENEQ TFS200 ALD) with a process temperature of 200 °C, resulting in a pinhole-free and conformal layer on the entire sample, including the backside, using adequate sample mounting on support silicon (Fig. 11c). The alumina passivation will serve as a hard mask during the vapour HF release step.

#### Alumina patterning

With the alumina covering the entire sample, certain regions must be opened up again: (1) within the MEMS cavities, so that the exposed buried oxide (BOx) sacrificial layer can be removed selectively to undercut the device layer silicon, and (2) over the metallization, so that proper electrical contact can be established.

The alumina is opened locally in two dedicated etch steps. The first etch step removes the insulating alumina on top of the metal contacts and the sealing rings (while the MEMS cavities remain protected by photoresist). The second step provides access to the waveguides in the regions for MEMS release (while the already opened metal areas remain protected by photoresist). The separation into two separate lithography, etc. steps results from the choice of an adequate etch recipe and compatibility with the underlying material.

As the alumina is directly deposited on the silicon layer also used for the waveguiding, a highly selective etch method only removing alumina but not silicon is needed. We use a liquid BHF etch step (90 s) to remove the alumina layer selectively on top of the silicon in the MEMS cavities (Fig. 11d).

However, liquid BHF would also attack the aluminium that is used in the metal contact pads and sealing rings. While the thickness of the aluminium is in principle thick enough not to be fully etched, BHF results in alteration of the surface, which impacts the electrical interfacing (solder-bumping, wirebonding) and the sealing (thermocompression bonding) downstream in the process flow. Thus, for opening of the metal contacts, a dedicated lithography and etching step by inductively coupled plasma (ICP, STS Multiplex, (2 × 45 s with 45 s pause in between to prevent photoresist burning) based on Cl_2_ /BCl_3_ chemistry is used (Fig. 11e).

#### MEMS release etch

We use an anhydrous vapour hydrofluoric acid etch (SPTS uEtch) for the release of the MEMS devices. Using etch rate calibration before sample processing, the vapour HF allows for controlled and stiction-free MEMS release. We use a recipe with low pressure (90 Torr) and 14 to 15 cycles of 900 s long etch steps, leading to a target average lateral undercut of 2.5 µm (Fig. 11f).

### Wafer-level hermetic sealing process

For the wafer-level transfer bonding of the silicon sealing caps, first, the sealing caps were fabricated on a separate 100 mm diameter SOI wafer. The layer thicknesses of the SOI wafer were 25 µm for the device layer, 1 µm for the buried SiO_2_ (BOx) layer, and 300 µm for the handle layer. The sealing rings of the caps were first patterned in the SOI device layer to be 5 µm high and 20 µm wide using photolithography and silicon deep reactive ion etching (DRIE). The sealing caps can be flexibly designed to feature dimensions from 450 µm × 330 µm to 2800 µm × 2950 µm. Next, a 100 nm-thick tungsten-titanium and a 2.1 µm-thick gold layer were deposited on the patterned device layer by sputtering. Next, I_2_/KI and NH_3_·H_2_O/H_2_O_2_ etching solutions were used in combination with a photoresist mask on the SOI wafer for pattering the gold and tungsten-titanium layers. Finally, the sealing cap outlines were patterned using a photoresist mask and DRIE of the 25 µm-thick device layer of the SOI wafer.

The completed sealing caps on the SOI wafer were then transferred to the silicon photonic MEMS device wafer by aluminium-gold thermocompression wafer bonding at a temperature of 250 °C. Therefore, the gold-coated sealing rings of the sealing caps on the SOI handle wafer and the corresponding aluminium rings on the PIC photonic wafer were aligned using a bond aligner (Suss BA8, Suss MicroTec AG, Germany) and clamped together on a bond fixture. Next, the bond fixture was transferred to a wafer bonder (Suss CB8, Suss MicroTec AG, Germany). Then, the chamber of the bonder was pumped to a vacuum pressure of <7 × 10^−5^ mbar, while the top and bottom wafer bond chucks were held at a temperature of 50 °C. The vacuum pumping was held for 60 min to outgas the wafer and cavity surfaces, thereby decreasing the gas residues inside the vacuum chamber and the cavities. Thereafter, the two aligned wafers were joined by applying a force of 28 kN by the bond chucks, resulting in a bond pressure (bond force divided by total sealing ring area) of 400 MPa on the sealing rings at the bond interface. While the bonding force was maintained, the top and bottom bond chucks were heated to 250 °C and held at 250 °C for 45 min, after which the temperature of the bond chucks was ramped down to 50 °C, with ramping times of 45 min (up/down). After the bond chuck temperature reached 50 °C, the applied bond force was released, and the bonded wafers were unloaded from the bond chamber. Finally, the handle layer of the SOI wafer was sacrificially removed using DRIE. This then leaves the individual sealing caps transferred and bonded on top of the cavities of the PIC photonic wafer, thereby encapsulating a vacuum atmosphere inside of the cavities and hermetically sealing the silicon photonic MEMS devices. Additional details on the wafer-level hermetic sealing process can be found in ref. ^64^.

### Interposer design and fabrication process

**Fig. 12:**
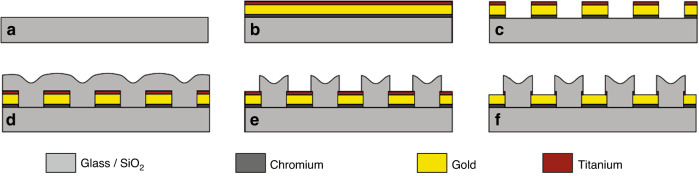
Schematic representation of the microfabrication process flow for interposer fabrication. **a** Starting glass substrate that is cleaned in a piranha bath, **b** chromium–gold–titanium metal sputtering, **c** maskless lithography and subsequent ion beam etch (IBE), **d** sputtering of isolation oxide, **e** oxide patterning by maskless lithography and buffered-HF liquid etch, and **f** titanium etch by IBE

We have designed and fabricated custom-sized glass interposers (typical area of 30 mm × 30 mm), which allow for redistributing the connections from the densely packed flip-chip contact pads (area: 50 μm × 50 μm, pitch: 150 μm) of the silicon photonic MEMS devices at the centre of the PIC to a wider pitch and routing them to larger, evenly spaced bond pads (area: 250 μm × 200 μm, pitch: 500 μm) at the edges of the interposer, which are compatible with standard wirebonding. The microfabrication process flow for the glass interposers is shown schematically in Fig. 12. First, a 100 mm glass wafer substrate is cleaned in piranha solution to ensure the absence of organic contaminants. Following this cleaning step, the substrate underwent chromium (15 nm)–gold (150 nm)–titanium (10 nm) deposition by sputtering. The chromium serves as an adhesion promotion layer between the glass and gold, and the titanium enhances adhesion between the gold and a subsequent oxide layer used to electrically isolate traces from one another. Following this initial metal deposition, the flip-chip contact pads, wirebond pads, and electrical traces are patterned using a maskless lithography step followed by an ion beam etch (IBE). Next, the insulation oxide (300 nm) is sputtered over the entire wafer and patterned to re-expose the metal in the contact regions (i.e., flip-chip and wirebond pads) using maskless lithography and a buffered hydrofluoric (HF) acid etch. After this liquid etch, an ion beam etch removes the titanium above the gold, ensuring that a proper electrical connection can be made in the subsequent solder- or gold-bumping process at the contact pads in the centre and in the wirebonding process for the contact pads at the edges, respectively. Finally, the 100 mm wafers are diced into individual 30 mm × 30 mm interposers.

### Electronic–photonic assembly by flip-chip bonding and fibre-array attach

**Fig. 13:**
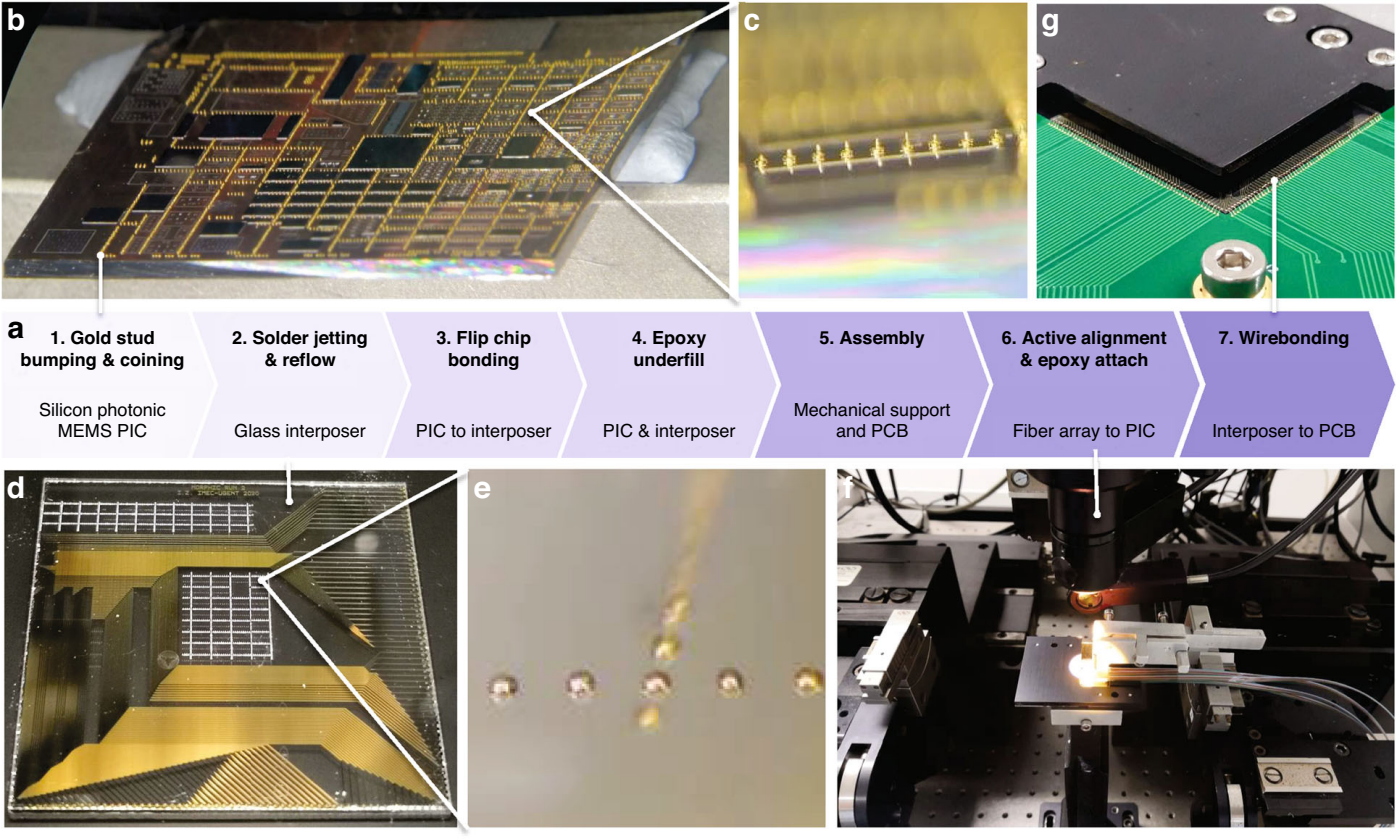
Electronic–photonic assembly process. **a** Flow chart for scalable electrical and optical interfaces by providing gold stud bumps on the PIC (**b**, **c**) for flip-chip bonding to a glass interposer with solder-jetted bumps (**d**, **e**), and subsequent active alignment (**f**) and epoxy attach of fibre arrays for the high count of optical interfaces, and finally wirebonding (**g**) to a printed circuit board (for the final assembly, see Fig. 9 in the main text)

The electronic–photonic assembly consists of seven individual integration steps, as schematically shown in the flow chart of Fig. 13a. Gold stud bumps are produced mechanically on the aluminium pads of silicon photonic MEMS chips using a Kulicke & Soffa (K&S) Icon plus LA thermosonic wire bonder. A 25 µm gold wire diameter was used for the bumping process for short and equal bump tail heights required for flip-chip bonding. In our case, 3400 gold bumps were placed on the silicon photonic MEMS chip, as shown in Fig. 13b. Since the wirebond stud bumps exhibit a residual wire tail height (Fig. 13c), a coining process was followed up after gold stud bumping by applying 35 N force to the pickup tool of the flip-chip bonding machine to ensure that all the bumps provided a flat bonding surface. SAC 305 (96.5Sn–3.5Ag–0.5Cu) lead-free solder balls of 50 µm were deposited on the gold pads of the glass interposer with the help of an SB^2^ laser solder jetting system by PacTech Ltd., as shown in Fig. 13d. The solder reflow process was carried out for 30 s at 250 °C to form metallurgical bonding between the solder balls and the bond pads on the glass interposer. The approximate height of these solder balls after reflow was measured at 30 µm, as shown in the close-up of Fig. 13e. The electrical connection between the MEMS device and glass interposer was established through the flip-chip bonding process. The silicon photonic MEMS chip was mounted directly onto a glass interposer ‘face-down’, precisely aligning the pad position of the MEMS device to the pads of the glass interposer, after which a temperature reflow process was performed for 30 s at 250 °C. Figure 9c shows the flip-chipped silicon photonic MEMS chip onto the glass interposer.

To further make a stronger attachment between the gold stud bumping and the solder balls, two-part underfill epoxy (EPO-TEK, 301-2) was inserted between the chip and the interposer and cured for 36 h at room temperature. A high-density PCB interconnection board was designed to connect the silicon photonic MEMS to external driver boards. We manufactured a custom mechanical assembly to package the silicon photonic MEMS chip rigidly. The glass interposer flip-chipped with the silicon photonic MEMS chip was bonded on the customized mechanical metal support with a room temperature curing Ag (silver) epoxy (Chemtronics, CW2400) and cured for 5 h. The mechanical metal support consisted of two parts: a top cover and a bottom plate. The metal support protects the silicon photonic MEMS chip and the glass interposer from external physical impact. Our silicon photonic MEMS chip contains 72 grating couplers to facilitate I/O coupling of the optical carriers to 72 matching PM fibres. A 72-channel fibre array was mounted on a 6-axis degree of freedom autoaligner system with a maximum resolution of 50 nm, as shown in Fig. 13f. A shunt waveguide connects the outermost grating couplers 1 and 72, which enables the precise alignment of the fibre array. We measured 10.4 dB loss from grating coupler 1 to 72 using a broadband light source. After precise alignment of the fibre array, optical UV epoxy (Dymax, OP-4-20632) was applied and cured for 60 s. The fibre array bonded silicon photonic MEMS chip and the interposer were mounted in a mechanical metal housing with a PCB. Finally, the pads of PCB and the glass interposer were wire-bonded by 25 µm pure gold wire, as shown in Fig. 13g. During the wire-bonding step, we observed that the 150 nm thin gold layer delaminated for some wire bonds, and a thicker gold layer is recommended to increase the reliability of this process step.

### Optical characterization of the MEMS-tunable building blocks

**Fig. 14:**
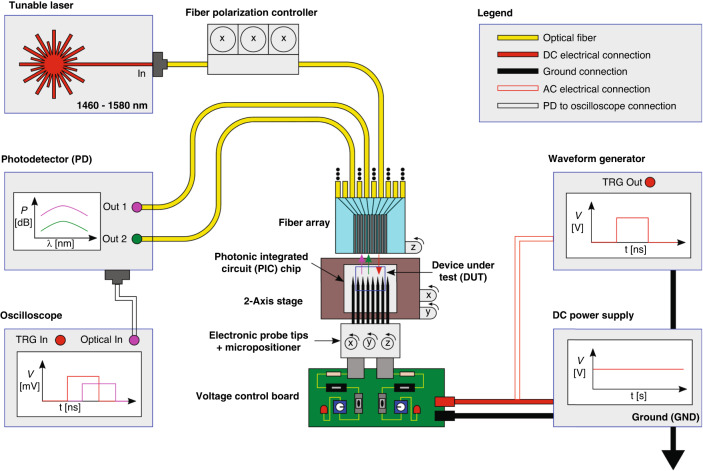
Schematic of a typical photonic MEMS test setup used here to characterize the power couplers and add-drop filter. Light with wavelengths between 1460 and 1580 nm is injected into a fibre array from a tunable laser and passes through a polarization controller to ensure propagation of only the TE mode. The fibre array is in turn carefully aligned with on-chip grating couplers using a combination of the 2-axis stage on which the chip rests and the z-control on the fibre array holder. Following the optical alignment procedure, actuation voltages are applied via a set of electronic probe tips whose position can accurately be adjusted using micropositioners. For DC measurements, the actuation voltage comes from a DC power supply connected to a custom-built voltage distribution board, and for transient measurements, this power supply is replaced with a waveform generator. Optical power measurements are recorded using the integrated photodetectors, which are connected to other fibres in the fibre array. If the signal should be observed in the time domain, the photodetector can be connected to an oscilloscope

Characterization of the presented MEMS-enabled silicon photonic devices requires the monitoring of optical power transmission over a range of wavelengths and actuation voltages. A schematic representation of a typical test setup configuration used for the characterization of our silicon photonic MEMS is shown in Fig. 14.

Light is injected into the chip by coupling the output of a tunable laser (1480–1580 nm) via a fibre array aligned to a set of grating couplers or using the attached fibre array for the packaged devices described in the previous section. The initial alignment procedure has the laser pass through a shunt connection on the chip such that it enters one grating coupler, passes through a section of integrated waveguides, and then immediately exits through another grating coupler into another fibre, where the power can be monitored by a photodetector. By maximizing the power transmission in this configuration, it is possible to accurately align the fibre array with respect to the devices under test. This procedure has the additional benefit of providing a baseline measurement for the grating coupler spectrum when needed, such as experimentally determining the insertion loss of the device.

Following the optical alignment, electrical contact with the metal pads can be made via the electronic probe tips that are mounted to a set of micropositioners. The actuation voltage is distributed to the various contact pads/probes using a voltage control board that is connected to either a DC power supply or a waveform generator. With each incremental change in the actuation voltage, the optical response is recorded by measuring the amount of transmitted power to the various outputs using separate photodetectors. If the response should be measured in the time domain, the optical signal can be converted to an electrical signal by a high-speed photodiode that is then sent to an oscilloscope.

When we characterize the power coupling devices, the optical power received at the drop and through ports is of interest. By performing wavelength sweeps from 1510 to 1580 nm with a 100 pm step size at each step increase in the actuation voltage, the transfer of power between outputs can be observed. We measured the waveguide switch with a similar setup as the power coupler. A difference was the additional actuation bias needed.

For the phase shifters, TBU, and add-drop filter, we also used similar setups with control over wavelength and actuation voltage, although with one additional requirement: those devices feature interferences, which need to be captured with a finer wavelength resolution. As a result, we used a 13 pm step size for the phase shifter and TBU and reduced the wavelength sweep to approximately 10 nm centred at approximately 1553 nm for the add-drop filter. With this arrangement, it is possible to inject optical power at the input port and then observe the steep troughs and peaks in the through and drop ports, respectively, at resonant wavelengths.

### Mechanical characterization of suspended MEMS

MEMS-tunable devices that rely on the displacement of movable structures require a release step to partially suspend the movable structures. As those structures are released, a mechanical deformation is typically observed due to the residual stress in the device layer, built-up because of the different thermal expansions of the different materials in the layer stack. This is a well-known problem in MEMS design, with known solutions such as folding springs to avoid buckling and dedicated test structures^67^. In the case of photonic MEMS platforms, the device layer is much thinner than for standard MEMS platforms, and the resulting lower out-of-plane stiffness of suspended structures makes it even more important to address the issue of residual stress.

**Fig. 15:**
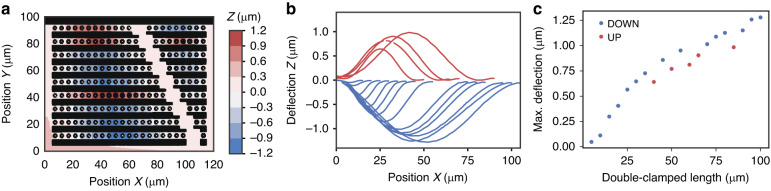
Mechanical characterization of double-clamped suspended beams. **a** 2D deflection map using an optical profilometer (Veeco Wyko NT9300). **b** Corresponding deflection profile of the different beams and **c** maximum deflection versus beam length

We implemented a series of test structures to investigate buckling caused by the release. In Fig. 15, we present the results for double-clamped beams with lengths ranging from 5 to 100 μm. The beams consist of the repetition of a 5 × 5 μm^2^ square, which was used to design the shuttles for the in-plane devices shown in the paper. The beams follow the expected deflection profile, with most beams buckling down (see Fig. 15b). Buckling is observed even for the shortest beams, highlighting the importance of avoiding double-clamped designs for in-plane photonic MEMS actuation (see Fig. 15c).

### Characterization of optical transitions and crossings

Five multimode interference (MMI) lengths ranging from 12.8 to 16.8 µm were designed to determine the length for which self-imaging occurs right at the crossing of two MMIs. Optical paths with 6, 18 and 30 crossings were designed for each MMI length. The fabricated chip was measured using a tunable laser and a power metre to determine the transmission response for wavelengths ranging from 1510 to 1580 nm. The insertion loss of the crossings was calculated for each measured wavelength by dividing the difference in transmission (∆*T*) by the difference in number of crossings (∆crossings) in the path, i.e., ${\textstyle{{{{{\mathrm{{\Delta}}}}}T} \over {{{{\mathrm{{\Delta}}}}}{\rm{crossings}}}}}$. Extracted wavelength-dependent loss values are plotted using a box plot to show the spread of the loss for the measured wavelengths (i.e., from 1510 to 1580 nm). For optical transitions, optical paths with 10, 30, 50 and 70 transitions were designed for each waveguide core width. The wavelength-dependent insertion loss was calculated using the same method as for optical crossings, i.e., ${\textstyle{{{{{\mathrm{{\Delta}}}}}T} \over {{{{\mathrm{{\Delta}}}}}{\rm{transitions}}}}}$.
