# Correction: Integrated silicon photonic MEMS

**DOI:** 10.1038/s41378-023-00649-2

**Published:** 2024-01-24

**Authors:** Niels Quack, Alain Yuji Takabayashi, Hamed Sattari, Pierre Edinger, Gaehun Jo, Simon J. Bleiker, Carlos Errando-Herranz, Kristinn B. Gylfason, Frank Niklaus, Umar Khan, Peter Verheyen, Arun Kumar Mallik, Jun Su Lee, Moises Jezzini, Iman Zand, Padraic Morrissey, Cleitus Antony, Peter O’Brien, Wim Bogaerts

**Affiliations:** 1 École Polytechnique Fédérale de Lausanne (EPFL), 1015 Lausanne, Switzerland; 2 School of Aerospace, Mechanical and Mechatronic Engineering, The University of Sydney, Camperdown, NSW 2006 Australia; 3 Swiss Center for Electronics and Microtechnology (CSEM), 2002 Neuchâtel, Switzerland; 4 Division of Micro and Nanosystems, KTH Royal Institute of Technology, 114 28 Stockholm, Sweden; 5 Department of Information Technology, Photonics Research Group, Ghent University - IMEC, Technologiepark-Zwijnaarde 126, 9052 Gent, Belgium; 6 imec vzw. 3DSIP Department, Si Photonics Group, Kapeldreef 75, 3001 Leuven, Belgium; 7 Tyndall National Institute, Lee Maltings Complex Dyke Parade, Cork, T12 R5CP Ireland

**Keywords:** Other photonics, Nanoscale devices, NEMS

Correction to: *Microsystems & Nanoengineering*

10.1038/s41378-023-00498-z published online 20 March 2023

After publication of this article^1^, it was brought to our attention that the author Iman Zand was omitted in the author list. The original publication has been corrected.

## References

[1] Quack, N. et al. Integrated silicon photonic MEMS. *Microsyst. Nanoeng.* **9**, 27 10.1038/s41378-023-00498-z (2023). 10.1038/s41378-023-00498-z PMC10025136 36949734

